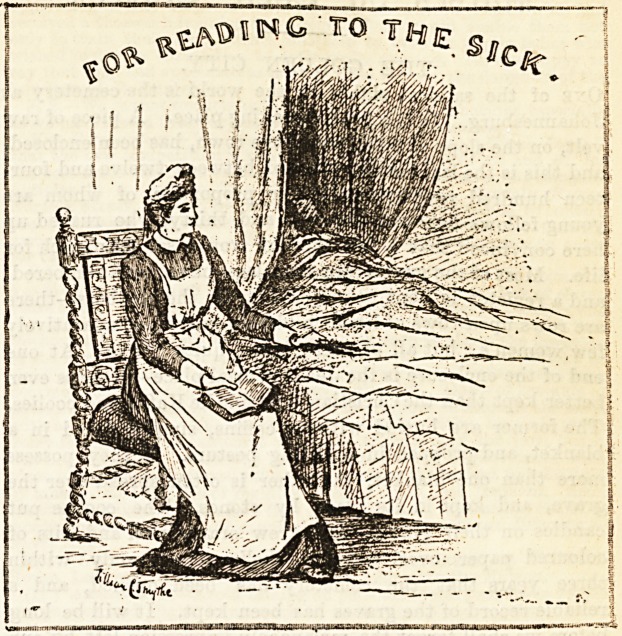# Extra Supplement—The Nursing Mirror

**Published:** 1890-11-22

**Authors:** 


### "The Hospital" Nursing Mirror.

Being the Extra Nursing Supplement of "The Hospital" Newspaper.

All Contributions for this Supplement should be addressed to the Editor, The Hospital, 140, Strand, London, W.C., and should have the word "Nursing" plainly written in left-hand top corner of the envelope.

### En Passant.

NURSES' INVENTIONS.—A bronchitis kettle, a bed-lift, and now a patent splint!—Our nurses are evidently increasing in inventive power. The new splint is called the trolly splint, and is only sold by the patentee. A narrow wooden tray is placed on the bed by the side of the patient; on this is placed the "Trolly Splint"; which moves on four small wheels. After the patient's arm is dressed, it is placed on the "Trolly Splint," he can then move his arm with ease. The nurse has used this splint with great success. It can also be used for a leg splint.

THE SPECIAL PRO.—The writer of the letters on "Six Months in a General Hospital" has come in for some very scathing remarks from some of our correspondents, and we ourselves are blamed for printing the letters. But is it not well to show forth all sides of nursing life? There was also the question of whether it was a good plan to have special probationers put in absolute charge of serious cases before they had had proper training. Is such a system good? That the letters were founded on fact there is no doubt whatever, and there was much to be learnt from them, if not in the line of what to do and say, still in the line of what not to do and say. We are glad, however, that the unsympathetic tone of the letters has been condemned by our readers.

CATHOLIC NURSES.—A correspondent sends us a number of the *Catholic News*, saying that as a Catholic nurse she was much pained at the tone of Miss Blennerhassett's remarks. The paragraph in the paper sent is as follows:—"I am more than gratified to see that Miss Rose Blennerhassett's rash and ignorant statements concerning what she was pleased to designate 'the incompetence, lethargy, and untidiness—(the very mildest word one can use)—of the Catholic nun-nurses' at Johannesberg have not passed away into the obscurity they deserve without a protest from Bloemhof, Transvaal. The writer of the defence attributes the lady's opinion to ignorance rather than to prejudice, and he contradicts it without fear or favour. The words she used are a 'shameful scandal' on an estimable body of pious and benevolent ladies,' whose 'charitableness and earnest devotion to the sick and needy of the goldfields' have been recently the subject of a highly eulogistic article in a Johannesberg paper. (I believe they are the Sisters of the Holy Family). There are hundreds of living witnesses on the goldfields to the competence, steadfast attention, and, above all, heartfelt sympathy of the Catholic nuns towards suffering humanity of whatever religious persuasion." We should have preferred to have had the authority on which this paragraph is presumably based. A mere denial is a poor defence, and we say this in all honesty, having no desire to enter into the religious question at all. Our columns have always been open to nurses of all denominations. Besides, we would point out that Miss Blennerhassett never was pleased to designate the Catholic nun-nurses by the phrases used above. Miss Blennerhassett's article appeared in The Hospital for August 16th, and we think it is rather hard on her that she should be called "rash and ignorant" when the rashness and ignorance certainly lies with those who give as Miss Blennerhassett's, words we emphatically state she never wrote.

THAT UNHAPPY COUNTRY.—In the *Londonderry Sentinel* for November 6th appeared a calm and practical letter from Miss Wilson, pointing out that as the guardians require certificated teachers for the workhouse children, so should they require certificated nurses for their sick. She also pointed out that economy was best achieved by a thoroughly good nurse. Well, a few days later the Limavady guardians met again, and passed the following resolution: "That at a special meeting of the guardians of the Limavady Union, called to consider the letter of the Local Government Board, and also the doctor's report, the guardians decided that they are satisfied with the manner in which the duties of infirmary nurse have been discharged since her appointment, and were of opinion that no change was necessary." It is surely time the guardians were enlightened by some doctor, or other competent person, as to their ignorance on nursing points; otherwise they will find themselves in the disagreeable position of being forced to give in to the Local Board, which has tried gentle hints in vain.

THE SICK NURSE.—Two cases of illness in nurses have lately come before us, which we cannot but regret as partly the fault of the sufferers. The first case is that of a nurse now in her thirtieth year, but who began work when only twenty. She was never very strong, and the matron or medical superintendent who passed her made a great mistake; that nurse is now a hopeless wreck, and though she attributes her illness to the special strain of trying once to lift a helpless patient, there can be no doubt that she was never fit for a nursing career. She might have lived a long and useful life in some easier path of duty. The other case is that of a monthly nurse, naturally troubled with insomnia; of course, monthly nursing was the very worst branch of the profession she could have chosen, for the monthly nurse often gets only three hours of unbroken sleep at a time for weeks together. The needs of the wee babies are so very frequent that it requires a special aptitude for swift sleep and waking in their nurses, and it was simple madness for a woman suffering from insomnia to take up this work. There is no merit in a lengthy suicide, and nurses ought to have the common sense to know this.

SHORT ITEMS.—The Metropolitan and National Nursing Association appealed on Thursday to the Council of the Hospital Sunday Fund for a grant.—Miss Coombe, the matron of the Hospital for Sick Children at Bristol, has held her present post for nine years; her services are greatly appreciated by the committee.—Some twelve English nurses are now working at Montreal under Miss Gee, and are giving great satisfaction to the doctors.—Mrs. Ormiston Chant lectured at Hammersmith last week on "Notable Women," and said of Miss Nightingale that she, underneath the heavy burden of a cross of pain, had opened up a new field for the highest types of women as the emancipators from pain.—The *Lancet* of last Saturday had an excellent note on the Midwives' Registration Bill. It said: "The whole effect of the proposed legislation is to make the assumption of the title of midwife more difficult than it is now, and to reduce the numbers of a class already too numerous."—The ladies' committee of the Establishment for Gentlewomen having dismissed Miss Meyrick without cause, the gentlemen's committee and the medical staff have resigned, and the institution is closed.—Princess Beatrice visited the Edinburgh Jubilee Institute last week, and was shown round by Miss Guthrie Wright.—Miss J. M. Bennett, and other sisters of the Indian Service, sail in H.M.S. *Malabar* on December 11th.

### Lectures on Surgical Ward Work and Nursing.

By ALEXANDER MILES, M.B. (Edin.), C.M., F.R.C.S.E.

LECTURE IV.—ANTISEPTICS IN COMMON USE—(*continued*).

(1) IODOFORM.—This substance is practically a preparation of iodine, and is met with in three forms. (1) Crystals, large, irregular, rough, and coarse; (2) powder, which is simply these large crystals crushed and broken down into small, regular, golden-yellow particles; (3) Sublimated powder, which is a fine flour-like impalpable powder.

The last-mentioned is the best form to use. In whatever form it is, it has a peculiarly persistent and somewhat disagreeable odour. This disagreeable odour may be masked, or at least altered, by various means, such as tincture of musk, tonquin bean, balsam of Peru, and so on. It is said to have an anodyne action when applied locally, and has been used in painful affections of the rectum, as a suppository, on this account. It is a fairly powerful antiseptic, and is said to have a specially beneficial action in all tubercular affections, and also in certain venereal diseases. It is used largely to powder over operation wounds and at subsequent dressings.

In all conditions in which the discharge has a disagreeable odour, such as septic abscesses, open cancers, and so on, iodoform is a very valuable application, its own odour serving to mask that of the discharge.

Carbolic or plain gauze is often charged with iodoform, and used as a deep dressing, or to stuff cavities. For the latter purpose one long strip of gauze is preferable to a number of shorter strips, as it is more easily removed, with no risk of leaving any in. Iodoform should be kept in a cool, dry place.

CAUTION! In children, and in old weakly people, symptoms of iodoform poisoning are said to occur when large quantities have been used, and especially if the powder has been blown into cavities and left there. These symptoms vary much, and differ in the young and old. It seems quite probable that the symptoms assigned to iodoform absorption are really those of other complications entirely. They are, loss of appetite, mental depression or excitement, and sometimes more grave brain symptoms. Should these occur the drug must be discontinued and the patient stimulated.

ADVANTAGES.—(1) General applicability; (2) general efficiency; (3) special action in tubercular and venereal diseases; (4) deodorising properties; (5) anodyne properties.

DISADVANTAGES,—(1) Its expense; (2) its persistent odour; (3) alleged poisonous properties.

USES. (1) To dust on all wounds, especially tubercular and venereal. (2) To dust on the conjunctiva in purulent conjunctivities. (3) To dust on to the peritoneum in laparotomy. (4) As an insufflation for nose, ears, rectum, etc. (5) To charge gauze for stuffing cavities, etc. (6) As a deodoriser, *e.g.*, in cancerous ulcerations. (7) In erysipelas.

PREPARATIONS. (1) Iodoform powder. (2) Iodoform ointment. (3) Iodoform suppositories. (4) Iodoform wool. (non-officinal). (5) Solution in ether. This is painted on to the wound, and when the ether evaporates a coating of iodoform is left.

ANTISEPTIC UNGUENTS.—In spite of the advances which have been made in the preparation of antiseptic agents, we still seem to be in want of a satisfactory antiseptic unguent or oil. Those in ordinary use for the lubricating of catheters, bougies, exploring needles, etc., have each some disadvantage; some are irritating, and others are unreliable as germicides.

(1) CARBOLIC OIL consists of 1 part of carbolic acid dissolved in 5, 10, or 15 parts of olive oil, according to the strength required. Although this preparation is often recommended for antisepticising catheters, hypodermic needles, etc., it is by no means a certain agent. "The value of these oily compounds is very doubtful, as they have been found to have no influence on germs" (Mitchell Bruce). When freshly prepared it may be efficient, but it has been shown that, after standing for a short time, it loses all its germicidal power and is simply a plain oil. I have tested a large number of samples of so-called carbolic oil found in various hospital wards, and have almost without exception found them to consist of simple olive oil.

You should, therefore, never use carbolic oil to disinfect instruments unless it is perfectly freshly *prepared*; to be fresh from the shop is not sufficient, it may have stood there long enough.

(2) EUCALYPTUS OIL, or eucaliptol, is the oil distilled from the fresh leaves of the plant of the same name—a species of gum tree. It is of a pale straw colour, and has an agreeable aromatic odour. It is a fairly reliable antiseptic, and on this account, as well as because it is less irritating, it is preferable to carbolic oil.

(3) An unguent, of which the following is the recipe, Rx. vaselini: Olei vaselini, āā ℥iip; cocain, 4 per cent.; olei eucalypti, ʒi., has been found very useful for lubricating urethral instruments. It is a good antiseptic lubricant, and the cocaine seems to sooth the urethral mucous membrane, preventing subsequent spasm and urethral fever.

*(To be continued.)*

### Examination Questions.

PRIZE ANSWER FOR OCTOBER.

IT is important that a nurse should maintain her general health, avoiding all causes that tend to lower the system. She should live well and take no stimulants. Daily exercise in the open air is necessary, but not so as to over-fatigue herself. A sufficient amount of sleep must be obtained. Avoid inhaling the patient's breath, or odour from the excreta. It is well after attending to the patient to rinse out the mouth and nostrils with a weak solution of Condy's fluid. Some strong disinfectant should be placed in the utensils used for receiving the fæces. The room should be ventilated by opening the window at night as well as daytime, care of course being taken to protect the patient from all draught. A fire in a room greatly assists ventilation. The nurse should keep as much as possible between the window and the patient and thus breathe the fresh air. She must wear washing dresses and take a daily bath. For the safety of others, those suffering from typhoid fever should be separated from the household or other individuals, and only those who have business in the sick-room should be admitted. Although articles of furniture cannot carry the infection, unless contaminated by the stools, it is well to remove unnecessary articles, as it admits of greater cleanliness and air. The patient, the patient's bed clothes, and room, should be kept scrupulously clean. All soiled linen should be placed in a disinfectant fluid before leaving the room. If possible, a separate water-closet should be used for all the excreta of the patient, and flushed daily with disinfecting fluid. In the country the doctors sometimes expect the nurse to bury all matter capable of sowing infection in some far corner of the garden. The air of the room should be impregnated with some disinfectant, carbolic acid, or other volatile material. A sheet saturated in dilute carbolic acid should be hung over the doorway. All food that has been in the sick room should be thrown away. Inquiry should be made as to the drainage of the house, also the water supply and the milk. The room and its contents must be thoroughly disinfected when the case is ended.

### The Nurses' Bookshelf.

### ASYLUM ATTENDANTS.*

This excellent handbook was prepared by a sub-committee of the Medico-Psychological Association, and is a clear and concise guide to the duties of asylum attendants. The chapters are: (1) The Body, its general functions and disorders; (2) the Nursing of the Sick; (3) Mind, and its disorders; (4) the Care of the Insane; (5) General Duties of Attendants. The paragraphs are side-headed, and there is an appendix consisting of a list of all the noted asylums and the names of their medical superintendents. We have before quoted very fully from this book, but we cannot resist repeating one short passage again: "Attendants should not make a promise to a patient unless it is to be fulfilled. They should try to win the confidence of the patients, by sympathy, kindness, and due consideration for their feelings. They should not hold themselves aloof from their charges, or be content with supervising them, but should join heartily in their occupations and amusements, and work both with and for the patients." The writers of the handbook are very practical in all their suggestions, and yet they seem to sympathise with the trials of the attendants, and to know how much of asylum labour can never be expressed by rule. The evil habit of regarding the insane as incurable is especially prevalent amongst attendants and destroys their interest in their patients. All asylum attendants and all private male nurses should study this book with care, and thoroughly master the contents. The price of the book is not mentioned, but we should think it is not more than half-a-crown.

### A ROYAL EXAMPLE.†

There are some passages of this book which must interest all nurses, for the Princess of Wales has ever been a noble example to all women in the sympathy she has shown with suffering. On page 147 is an account of the opening of the Marylebone Infirmary by both Prince and Princess, and on the next page the story of the start of the Lady Augusta Nursing Institution, in which the Prince took part. The opening of the Nurses' Home at the London, the reception of the First Thousand at Marlborough House, the opening of the Royal National Hospital, and of the Mary Wardell Home, and many more accounts of functions of interest to nurses will be found in this volume.

### A PRACTICAL HANDBOOK.‡

Many of our readers must be already well acquainted with such of Dr. Lewis's lectures as have appeared in our pages, and from the warm appreciation they then received, we are sure they will be welcome in a more complete form. The lectures have received several additions, and are furnished with questions and a full index; as a cheap and handy book for the young probationer this volume is unrivalled; it ought also to prove of great use to amateur nurses. It has only one want, and that is illustrations; but, then, you cannot get everything for a shilling. Dr. Lewis writes very clearly, and is always much to the point; he does not carry the young nurse too far afield, or make any attempt to teach her medicine; still, there are special chapters on chest diseases, artificial feeding, tracheotomy, treatment of restlessness, delirium, the eye, and ventilation. The book is nicely got up; good print and neat binding, and ought to be useful as a Christmas present.

* Handbook for the instruction of Attendants on the Insane. (Bailiere Tindall, and Cox)

† "Prince, Princess, and People." By H. C. Burdett. People's edition. Price 1s.

‡ "The Theory and Practice of Nursing," by Percy G. Lewis, M.D. Price 1s. Published by THE HOSPITAL (Limited), 140, Strand, W.C.

### TO HOSPITAL PATIENTS.

BY looking at sickness in a practical light, we may perhaps reach some hearts which would be untouched if we spoke to them about religion. This is a very sad thought to those who truly love God, but as St. Paul tells us to be "all things to all men in hopes of saving some," we will just think of the advantages we gain from being loving and contented with what falls in our way.

For instance, people soon get tired of listening to complaints and grumbling, and if we never have a cheerful word or a smile for those who attend on us, we can't expect they will come near us more than they can help. Besides, it is a deal wiser to try and remember what a number of persons and things they have to look after, and how wearisome it must be, day after day, to see nothing but sick people on all sides. When we have got this spirit of forbearance in our hearts, a few kind words or a smile go a long way to cheer the day, and make us feel we are among friends.

Then another good way to make our lives happier is to try and get up an interest in other people's lives. Those who are in a hospital ward have plenty of opportunities of hearing about their neighbours' woes. They can compare their illnesses, and see which has the best of it, for it is often a great comfort to find that others have more to put up with than ourselves, and by pitying them we soften our own hearts and become happier. But it would be still wiser to get away from ourselves altogether, or as much as we can, and think and talk of husband or wife or children, or those dearest to us, and if our companion does not care to hear about them, then just listen to his or her story; you will make new acquaintances as it were, and you may get a lesson by thinking in your own mind whether you would have behaved as well if you had been tried as much as they were.

But whatever you do to divert your minds let it be done in a kindly spirit, with an eye always on pleasing others, and making them happy. We know of One who pleased not Himself; but we are so fond of ourselves, and love ourselves so well—as indeed is only natural—that if we do not take care we shall simply become selfish. How we hate to see other people wrapped up in themselves, struggling for the warmest and most comfortable place, scheming to get the best and choicest morsel from the dish! Let us each one try not to fall into such ways. There was one Man who suffered hunger and thirst, who was even put to death, who bore all and everything without a murmur, who when He was reviled, reviled not again, who was dumb before His accusers as a sheep before its shearers.

### Nurses on Their Travels.

### THE GOLDEN CITY.

One of the saddest sights in the world is the cemetery at Johannesburg. It is a heart-breaking place. A piece of raw velt, on the slope of a hill above the town, has been enclosed, and this is the last resting-place of between twelve and fourteen hundred people, the larger proportion of whom are young fellows between nineteen and thirty, who rushed up here convinced that a few months would see them rich for life. Most of the graves are nameless, but all are numbered, and a register is kept corresponding to the numbers—there are rows upon rows of little children's graves, comparatively few women's; but all of these latter quite young. At one end of the enclosure is the Jewish burial place—perhaps even better kept than the Christian—outside lie Kafirs and coolies. The former are buried without coffins, simply rolled in a blanket, and propped in a sitting posture. If they possess more than one blanket, the other is often spread over the grave, and kept in its place by stones. The coolies put candles on their graves, and strew orange-peel and bits of coloured paper over them. I believe it is only within three years that the cemetery has been opened, and a reliable record of the graves has been kept. It will be long before we shall forget the melancholy impression left by our visit to it. Just beyond the cemetery is a glittering mountain. When the sun shines on it, it sparkles with a thousand prismatic colours, and looks like the entrance to the palace of diamonds, where the fairy Florizella lives; but in reality it is merely the place to which all the rubbish of Johannesburg is carted, and as apparently half the food of the town comes out of tins, the result is a meretricious splendour quite in keeping with the other attractions of "Goldopolis." I have been down two gold mines, and was, of course, much disappointed. To an outsider the long dirty underground passages seem very uninteresting, and it is impossible to realise that the wheel-barrows full of rubbish that Kafirs are driving along a sort of underground tramway are full of gold, and immensely precious.

But a battery or gold mill is exceedingly interesting; there is so much that an entirely ignorant person can see and understand. You see the rubbish out of the wheelbarrows emptied into funnel-shaped openings, through which it slides under the crushing machines, the steam hammers of which rise and fall with a deafening noise and a most attractive regularity. When the quartz is reduced to fine powder it escapes from the machines, and slides over tables covered with quicksilver, with which the gold unites, and forms what they call amalgam. Some of the gold escapes, and passes on over other tables, and finally collects itself into a muddy, sandy mess, which one would expect to see thrown away, but which is called "tailings," and is extraordinarily valuable, considering its unprepossessing appearance; it is afterwards treated chemically. From the mill we went to the gold-smelting and assaying office. The amalgam is put into a sort of retort; when heated, the mercury escapes, and dirty, black, spongy-looking stones remain. These, to our amazement, were said to be pure gold. From the retort they were put into a smelting furnace, in which they turned bright yellow, and went into brick-shaped moulds. A 500-oz. mould contains £2,000 worth of gold, we were told. The assayer showed us various interesting chemical tests, and we were delighted with his scales. He pulled out one of his hairs and showed us how it influenced the scales; and he told us that in weighing gold, a mistake of less than a-third of an ounce would make a difference of about £9,000 in the course of a month. If one remembers that everything in Johannesburg, from the great steam machines to these fairy-like weights and scales, have been dragged up from the coast by oxen, over hundreds of miles of wild country, and that, in the short space of three years, one is forced to acknowledge that the town, with its mines and gold mills, and its phantom of civilisation, is a very wonderful monument of human energy and pluck. And if the Johannesburgers would have the whole of the rubbish and refuse cleared out of the public places, would wrestle with the dust, and give Christian burial to the remains of oxen, horses, &c., which lie about in various stages of decomposition till the vultures have picked their bones clean, their city would be about the healthiest in the world, as well as one of the most curious.

### Everybody's Opinion.

[*Correspondence on all subjects is invited, but we cannot in any way be responsible for the opinions expressed by our correspondents. No communications can be entertained if the name and address of the correspondent is not given, or unless one side of the paper only be written on.*]

### SIX MONTHS IN A HOSPITAL.

"Nurse W." writes: Will you allow me a small space in your popular paper in which to say how glad I was to see in last week's issue a "protest" against the tone and spirit of "A Special Pro.'s" letters? If The Hospital were circulated solely amongst nurses, I should scarcely think such a protest necessary, as the letters depict a type of nurse probably known to most of us, and one, I fear, which is no credit to our profession. But knowing that your paper has a large circulation amongst people who have no experience of life inside a hospital, it is scarcely fair that such letters should be allowed to pass without a word from some of us who have been longer in the work. I am glad to say there are few nurses who take upon themselves the responsibilities and duties involved in caring for the sick and dying in such a flippant manner, and I quite agree with Miss Bayley in saying that if I were ill I should not care to be nursed by the "Special Pro" who wrote those those letters, though she is not a fair sample of special pro as I have known them. I should like to commend to her perusal a manual for hospital nurses, entitled "Our Work for Christ," by A. E. Morrell, and published by Rivingtons. [We have no room to publish other letters on this subject.—Ed.]

### MALE NURSES.

"J. E. C." writes: It is quite a delusion to believe that the Hamilton Association alone trains male nurses. Men are thoroughly trained to nurse mental and epileptic cases in every asylum in England, and it is for these cases only that male nurses are in much demand. In their last report the Hamilton Association says they have 50 nurses on their staff, and only 35 of these were engaged at the time. Surely this fact alone points to some fault in the rules or the administration?

### POST AS ATTENDANT.

Miss Wilson writes:—May I ask you to make known through your columns the following need: If any of your readers require an attendant for an invalid child, or delicate lady, I should be glad to recommend one of our Workhouse Infirmary Nursing Association probationers, who has during her training developed slight lung mischief. She is otherwise quite able to take charge of a light case. The doctors who have examined her say that a winter in the Engadine, or in some sunny place, like Hastings, Bournemouth, or Torquay, would entirely cure her. I shall be glad to give further particulars. Address, 6, Adam-street, Strand, W.C.

### PRAYER BOOKS FOR PATIENTS.

"Sister," writes: Since first entering hospital I have remarked how few Roman Catholic patients there are who are not only possessed of their beads, to remind them of their devotions, but also of little books of prayer; small, that any of them can hold it without being overcome with weakness in too short a time, and in type not so small that any ordinary eyes would ache too soon. It has so often been forced on my mind, the opportunity they have for drawing their minds from their present sufferings to things above, while protestants, I am sorry to say, seem not to have the same encouragement. A few days ago a patient showed me, with such pleasure, the prayer book "the father" had given to her, one in a bright blue cover and gold letters, with her name neatly written on a small gummed ticket, pasted on the outside; while I stood admiring it, to the great delight of its owner, I felt grieved when the poor woman in the next bed said, with a voice that sounded as if coming from the grave, for she is in the last stage of phthisis, "I wish Protestants got prayer books, sister." Certainly there are Bibles and often Church of England prayer-books supplied to the wards, of good-sized type, and substantial binding, consequently large and heavy—too heavy, indeed—for the majority of patients to hold. Many patients have lain on their backs for over ten, some for nearly twenty, years, and they have too little strength in their arms to hold these heavy books; but if they had a small book, with selections from the Bible and some prayers, what comfort they would have, not only in the reading of it, but in knowing it was their very own to read when they could, and to put under their pillow (for that is the patients' storehouse unless the nurse keeps a good look out) when they go to sleep. I am sure that many Protestant nurses have felt as I do on this subject, and I have waited long to see if any one better able to write would do so, but have seen or heard of nothing having been written, and feel compelled to do it myself, hoping some good man or woman will either write or compile convenient prayer-book for Protestant patients in hospitals.

### ASYLUM ATTENDANTS.

"TWO MENTAL NURSES" write: "We own there may be some that are not all they should be; but there are good and bad in all stations of life, and doctors connected with asylums should do all in their power to encourage rather than slander us in public periodicals. We have lived in county and private asylums, and find that county asylum nurses are equally as bright, educated, and refined as those in a private institution. Neither do we think a 'picturesque uniform' has anything to do with a refined mind or the benefit of those placed under their care. Let us hope the time is not far distant when asylum nurses will be better paid, and as highly appreciated as our sisters, viz., hospital nurses. We quite agree with the help of a good matron the nursing staff is greatly benefited, and the majority of head attendants and matrons are chosen from what Dr. Farrar chooses to describe 'a very low class of girls,' viz., the nurses."

"A 'HOSPITAL' READER", writes:—Having seen much lately in THE HOSPITAL respecting asylum attendants, I beg to differ from some of your correspondents in their opinions respecting them. Perhaps worthy medical practitioners, after arriving at the conclusion that they are a very low class of girls, believe they have performed the whole of their duty to society. Might not this wrong be righted by superintendents and committees of asylums being more choice in their selection of nurses? But if such statements, which are not altogether correct, are left undisturbed, who can expect respectable women to enter such situations? And if any one class of afflicted beings more than another need respectable intelligent attendants, surely the insane do who are so entirely shut out from the world. In this asylum no training is given. Could not something be done for the training of county as well as private asylum nurses? Would this be too trivial a question for the consideration of the County Council?

### NURSES' WATCHES.

"LINCOLN" writes: On reading "Practical Hints for Nurses" in THE HOSPITAL of Oct. 25th, I thought that hint a very good one about nurses using silver watches with seconds hand. I also had found the same difficulty with a gold one, and had obtained a nice sized silver watch with seconds hand. Others of my nurse friends have done so. We obtained them of Mr. G. W. Pament, practical watchmaker, 178, King Street, Hammersmith, London. I had thought of one with centre seconds, but was told it would be too large. Mine only cost 30s., and goes well.

### Notes and Queries.

TO CORRESPONDENTS.—1. Questions may be written on post-cards. 2. Advertisements in disguise are inadmissible. 3. In answering a query please quote the number. 4. If a private answer is desired, a stamped addressed envelope must be forwarded.

NOTICE.—We must really ask our correspondents to be more particular in enclosing name and address. Constantly we receive queries or letters with no name attached.

### Queries.

(12) *Temporary Home Wanted.*—Can any one tell me of a home for slight mental cases, where a young married woman could be taken for a time? There is hope of speedy improvement, if only she could have proper management. Can pay a little weekly.—*Hopeful.*

(13) *Starting a Home.*—Will any nurse or superintendent inform a certified nurse and lady, wife of a medical man, with a well-furnished house, the best way to form a small private, self-supporting Nursing Home? Have had experience with single patients.—*Thyrza.*

(14) *Asylums in India.*—Information required about private or other asylums in Madras or any other part of India.—*A.B.*

### Answers.

(8) *Diet.*—I advise K. G. A. to get "Diet for the Sick," by Dr. Ridge. Price 1s. 6d., published by Churchill.

*Miss Capewell.*—The following paragraph is from the *Birmingham Post* of October 18th, so that our information is correct, and yours is wrong: "My Lord explained that the committee, after careful consideration, had decided to reorganise the internal arrangements of the hospital, appointing a lady dispenser and securing as matron a lady who had received a thorough training in nursing. This would enable them not only to train their own nurses, but also to take as pupils ladies who wished to devote themselves to orthopædic nursing. He was happy to say that they had appointed as matron Miss Burges, the daughter of the late Rev. H. B. Burges."

J. S.—We cannot undertake to return rejected contributions, especially as we have stated that our poets are now far too numerous.

*New Competition.*—We beg to inform "Doris" and "Nurse Joyce" that the new competition is simply an offer of prizes of £2 and £1 for the best-made and most comfortably-shaped bed-jacket or nightingale. Articles to be sent to The Hospitals Association, Norfolk House, Norfolk Street, Strand, by December 17th, and each parcel to contain the full name and address of the sender, and a statement that the garment is her own make. We should like one hundred nurses to enter for this competition.

(9.) *Tetanus.*—I shall be glad to furnish "J.G." with the history of a case of tetanus which fully recovered. The patient was a boy of eleven years of age, who received a violent kick from a donkey. Tetanus was caused from the effects of the blow. When the child was brought into the hospital, the mouth was just sufficiently open to allow the insertion of the handle of a small spoon between the teeth. Frequent attacks of rigidity occurred, when the patient became purple in the face, the back became arched, and the limbs grew perfectly stiff. Doses of bromide of potassium were administered every four hours, poured through the teeth with a small spoon. The patient was fed by nutritive enemata given every four hours. An attempt was made to prise open the jaw, but it was not successful. After the first week a marked improvement took place; the jaw had relaxed sufficiently to allow the patient to take fluid by mouth, and the fits of rigidity became much less frequent. Soon bread and milk and other soft food could be taken, and at the end of a month the patient could open and close his mouth without difficulty.—*Nurse May.*

*Convert.*—There is a Catholic Nursing Association at Ladbroke House, Ladbroke Road, Noting Hill, W. Address to the Matron.

(11.) *Amusements.*—Avoid talking on personal topics; engage in games and puzzles and slight housework if in wards. Keep regular hours, give exercise, and be bright and chatty yourself.—*Scot.*

### Appointment.

[It is requested that successful candidates will send a copy of their applications and testimonials, with date of election, to THE EDITOR, The Lodge, Porchester Square, W.]

JAFFRAY HOSPITAL, BIRMINGHAM.—Miss Ada C. Bloomer has been appointed Sister of the male medical wards of this hospital. Miss Bloomer was for a short time at the Manchester Royal Infirmary, and for the last two years has been working at the Seamen's Hospital, Greenwich. We congratulate Miss Bloomer, and hope to chronicle her further success in the future.

### Vacancies.

THE following vacancies are announced:

### Matron.

Montrose Royal Asylum.

### Nurses.

Abbey Parochial Hospital, Paisley; £25.

Accident Hospital, Poplar (night).

Alexandra Hospital, Brighton; £25.

Bath Royal United Hospital; £25.

Blackheath and Richmond Institus.

Barony Parochial Hospital, Glasgow (night); £24.

Bournemouth Institute; £25.

Carlisle Union (supt.); £50.

City of London Chest Hospital; £20.

Canterbury Institute.

Devonshire Hospital, Buxton (head); £30.

Dewsbury Infirmary; £24.

Dorset County Hospital, Dorchester; £22.

Glasgow Sick Poor and Nursing Association; £25.

General Nursing Institute, Covent Garden.

Harding Institute, Stoke-on-Trent.

Huddersfield Infirmary; £26 12s.

Huddersfield Home.

Hull Home.

Islington Workhouse Infirmary; £20.

London Throat and Ear Hospital, W.C.; £14.

Liverpool Eye and Ear Hospital; £22.

Leicester Institution; £25.

Middlesboro' Association.

Newport (I.W.) Institute; £25.

Pontypridd Union; £27.

P.M.V. Homes, Addlestone, Surrey; £15.

Paddington Infirmary (night); £20.

St. Bartholomew's, Chatham (assist.); £15.

Sheffield Dispensary; £22.

St. John's Home, West Kirby.

Taunton and Somerset Hospital Institute; £18.

Victoria Home, Bournemouth; £25.

Workhouse Infirmary Association.

Worcester City and County Institn.

Worthing Institute; £25.

Western General Dispensary; £25.

Wilson Institution, W.; £30.

### District Nurses.

Jersey Institute; £25.
Middlesboro' Association.

### Probationers.

Beccles Cottage Hospital.

Bedford Infirmary.

Glasgow Cancer Hospital.

Glamorganshire and Monmouthshire Infirmary.

Liverpool Eye and Ear Hospital.

Middlesboro' Association.

Rugeley Hospital, Staffs.

Royal Hospital, Weymouth.

Workhouse Infirmary Association.

### Our Understandings.

It is a far cry from the first sandal of wood or raw skin to the dainty *brochure* of the fair lady of to-day ; or to the tiny pair of baby shoes, which are taken out surreptitiously from their hiding-place, to be gazed upon lovingly, perhaps kissed on the shabby toes, all worn and torn from the incessant swaying of the little feet that once inhabited them. Curiously interesting would be a procession of man's understandings, showing their gradual evolution out of the primary idea, the necessity, that is, for a foot protection against life's stony ways. Though for the matter of that, had man been sufficiently persevering in doing without foot coverings Nature would certainly have helped him, and all who came after him, by providing his tender soles with hereditary skins of horny consistency. Indeed, it is a fact that, at present, the soles of the very few North Country-folk who tramp barefooted over rock and heather, are found, upon examination, to be of adamantine hardness.

It is rather late in the day, however, for the nineteenth century foot to begin the experiment of sole hardening, consequently there is nothing for it but to carry on the process of improving the shape of foot-gear, as we go on improving—or thinking we do—other things.

Benedict Sabbouth, a wise man of the sixteenth century, and a shoemaker, to boot, wrote a treatise tracing the shoemaking of the ancients back to the mother of us all, who, after the manner of many a mother of our own days, harassed herself to provide foot-coverings for the children—those first little toddlers of our race who stumbled along life's uneven path. That's as may be ; it is possible Eve did naturally essay to invent some protection for those tender little feet.

"Without going back to Eden," says an American periodical, *The Boot and Shoe Journal*—and why should not our understandings have a periodical devoted to themselves when such countless numbers are provided for the understandings at the other extremity of man, his brain, that is to say?—"let it suffice to know that sons of St. Crispin can 'smile at the claims of long descent.' No doubt there was a time when all our forefathers went barefooted. In those good old days a man could step on his neighbour's toe without bringing a spasm of agony, for corns and bunions were unknown. But it has been discovered, by paintings on the walls of Thebes, that shoemaking formed a distinct and no doubt a lucrative trade away back in the reign of Thothemes III., some 1,500 years B.C."

At first, every man was his own shoemaker. In the early attempts at shoemaking the aim sought was not a covering for the foot, but a protection to the soles from sticks, stones, &c. The Egyptians made theirs of the bark of the papyrus, a rush growing on the banks of the River Nile. Of course it did not take long to find out that the sandals might be improved by "stitching a low rim or wall of leathering along the sides, and about the heels of sandals ; to these the straps or thongs were attached." By slow degrees, for invention creeps with leaden feet, these rims grew higher ; at last, they met, and, behold, there was the first shoe, crude and ungainly, but, nevertheless, a shoe.

*(To be continued.)*

### Where to Go.

Here are a few hints to the weary nurse who wants a change of thought and scene. Mr. Carrington Willis will give a Shakespearean recital at the Steinway Hall on November 25th. On Monday, Gounod's comic opera, "The Mock Doctor," will be revived at the Grand Theatre, Islington, where prices are cheap. This opera is founded on Molière's "Le Medicine Malgré lui," and Mr. Richard Temple will take the part of Sganarelle. At the Savoy Theatre "The Gondoliers" is being played every night at eight, and every Saturday afternoon at half-past two. The Arts and Crafts Exhibition at the New Gallery, Regent Street, is well worth a visit, admission 1s. There is a lecture every Monday evening at half-past eight. The British Nurses' Association will hold a conversazione on Dec. 5th : this is a side of the B.N.A. we heartily commend. On November 24th and 25th, General Booth will conduct services at Exeter Hall at eleven a.m., half-past two p.m., and seven p.m. ; reserved seats 1s., admission 1d. It may perhaps interest our readers to know that there are free public libraries at the People's Palace, Notting Hill, Brompton, Fulham, Hampstead, Kensal Town, Lambeth, Kensington, and Paddington.

### What to Read.

We hope all our readers know that they can now get a selection of Robert Browning's poems for a shilling. This neat little volume is published by Smith, Elder, and Co., but can be got at any bookstall. There is a little paper volume, "Senilia," or, poems in prose, by Tourgenieff, price one shilling, which is well worth reading. The poems are very short, and each gives food for much thought. "The Demoniac," by Walter Besant, is a study of the drink question, and written in a powerful and interesting manner. This also is published at one shilling. New novels to be had from libraries include "A Lost Illusion," by Leslie Keith ; "The Honourable Miss," by L. T. Meade ; and "The Word and the Will," by James Payn. Mr. Gilbert's "Songs of a Savoyard," and Mrs. Kroeker's "Gottfried Keller ; a Selection of his Tales," will repay perusal.

### Amusements and Relaxation.

### N.B.-THIRD QUARTERLY WORD COMPETITION

**Commenced Oct. 4, 1890; ends Dec. 27, 1890.**

Three prizes of 15s., 10s., 5s., will be given for the largest number of words derived from the words set for dissection.

Proper names, abbreviations, foreign words, words of less than four letters, and repetitions are barred ; plurals, and past and present participles of verbs, are allowed. Nuttall's *Standard* dictionary only to be used.

N.B.—Word dissections must be sent in WEEKLY not later than the first post on Thursday to the *Prize Editor*, 140, Strand, W.C., arranged alphabetically, with correct total affixed.

The word for dissection for this, the EIGHTH week of the quarter, being "NOVEMBER."

| Names. | Nov. 13th. | Totals. | Names. | Nov. 13th. | Totals. |
|---|---|---|---|---|---|
| Jenny Wren | 80 | 281 | Liz | 64 | 200 |
| Tinie | — | 55 | Checkmate | — | 76 |
| Agamemnon | 81 | 281 | Silver King | — | 163 |
| Patience | 81 | 281 | S. Anthony | — | 76 |
| Ecila | 81 | 281 | Quackah | — | 75 |
| Lightowlers | 74 | 267 | Reynard | 84 | 248 |
| Rouge | — | 89 | Sally | — | 27 |
| Wyamaris | 80 | 264 | Success | — | 61 |
| Qu'appelle | 80 | 259 | Caledonia | — | 52 |
| Nosam | 77 | 257 | Nurse Emma | 47 | 152 |
| Nurse Hilda | — | 44 | Hazel | — | 20 |
| Lady Betty | 82 | 265 | Pallas | — | 48 |
| Grenelle | — | 43 | Puss | — | 15 |
| Daisy | 76 | 245 | Shakespeare | 73 | 201 |
| H. A. S. | — | 157 | Melita | 76 | 261 |
| A. B. C. | — | 66 | Nora | — | 10 |

### Notice to Correspondents.

N.B.—Each paper must be signed by the author with his or her real name and address. A *nom de plume* may be added if the writer does not desire to be referred to by us by his real name. In the case of all prize-winners, however, the real name and address will be published.

Competitors can enter for *all* quarterly competitions, but no competitor may take more than *one first prize* during the year.

## Figures and Tables

**Figure f1:**